# Usefulness of “AcT ratio” in diagnosis of internal carotid artery stenosis: a multicenter, retrospective, observational study

**DOI:** 10.1007/s10396-024-01409-z

**Published:** 2024-04-06

**Authors:** Daisuke Tsukui, Hidehiro Takekawa, Kozue Saito, Ryuta Okabe, Akito Tanaka, Saro Kobayasi, Haruki Igarasi, Keisuke Suzuki, Hirotoshi Hamaguchi

**Affiliations:** 1 Stroke Center, Dokkyo Medical University, 880 Kitakobayashi, Shimotsuga, Mibu, Tochigi 321-0293 Japan; 2 Department of Neurology, Dokkyo Medical University, Tochigi, Japan; 3 Center of Medical Ultrasonics, Dokkyo Medical University, Tochigi, Japan; 4 Department of Neurology, Nara Medical University, Nara, Japan; 5 Department of Cardiology, Tokyo Metropolitan Tama Medical Center, Tokyo, Japan; 6 Department of Cardiology, Akiru Municipal Medical Center, Tokyo, Japan; 7 Department of Neurology, Kita-Harima Medical Center, Hyogo, Japan

**Keywords:** Acceleration time, AcT ratio, Measurement, Diagnosis, NASCET stenosis

## Abstract

**Purpose:**

The ratio of the internal carotid artery (ICA) to the common carotid artery (CCA), especially the “AcT ratio,” which is a modified measurement method of acceleration time, is useful for diagnosing ICA-origin stenosis. However, previous studies were single-center studies. Therefore, this multicenter, retrospective, cross-sectional study aimed to determine whether a method using the AcT ratio is useful for estimating stenosis rates.

**Methods:**

This study included 461 vessels subjected to carotid artery ultrasonography and evaluation for ICA-origin stenosis via NASCET at four hospitals. The duration from the steep rise point to the inflection point or the first peak was defined as AcT on pulsed wave Doppler. The AcT ratio was calculated as AcT of ICA/AcT of ipsilateral CCA. The AcT ratio and rate of ICA-origin stenosis were analyzed using Pearson's correlation coefficient, simple regression analysis, and ROC curve.

**Results:**

A significant positive correlation was observed between the AcT ratio and NASCET stenosis. NASCET stenosis of ≥ 50% had a sensitivity, specificity, and negative predictive value (NPV) of 70.2%, 71.6%, and 91.5%, respectively, when the cut-off value of the AcT ratio was 1.17. NASCET stenosis of ≥ 70% had a sensitivity, specificity, and NPV of 70.5%, 72.1%, and 95.9%, respectively, when the cut-off value of the AcT ratio was 1.22.

**Conclusions:**

The findings of this multicenter, retrospective, cross-sectional study suggest that the AcT ratio is useful for diagnosing ICA-origin stenosis, especially for diagnosis by exclusion. NASCET stenosis of ≥ 50% was considered unlikely if the Act ratio was ≤ 1.17, whereas NASCET stenosis of ≥ 70% was considered unlikely if it was ≤ 1.22.

## Introduction

Carotid endarterectomy and carotid artery stenting, in addition to strict medical treatment, have been recommended for the prevention of ischemic stroke and recurrence in patients with stenotic lesions near the origin of the internal carotid artery (ICA) [1, 2]. The North American Symptomatic Carotid Endarterectomy Trial (NASCET) method performed using digital subtraction angiography (DSA) has been recommended for patients with ≥ 70% stenosis [3]. Thus, it is important to evaluate the ICA stenosis rate using the NASCET method. For evaluations of NASCET stenosis, magnetic resonance imaging (MRI), computed tomography (CT), and carotid artery ultrasonography have been used in many hospital and clinics.

Carotid artery ultrasonography is a noninvasive and easy-to-perform examination that plays an important role in screening [4]. Carotid artery ultrasonography performed using pulsed wave Doppler (PWD) is useful, and the peak systolic velocity (PSV) is commonly used for estimation of the NASCET stenosis rate. However, measurement of PSV at the narrowest point is difficult in patients with a calcified lesion on the blood vessel wall. The presence of turbulence and prolongation of the acceleration time (AT) on the distal side of the calcified lesion indicate the presence of a stenotic lesion in such cases [4–6].

Takekawa et al. focused on the prolongation of AT and investigated whether estimation of the stenosis rate with a “modified AT” (AcT) is possible [5, 7–9]. AcT was found to be more useful than AT in estimating the stenosis rate in these studies. In particular, the “AcT ratio,” which is the AcT of the ICA divided by the AcT of the ipsilateral common carotid artery (CCA), was found to be the most useful [9]. However, these studies were conducted at a single institution. Therefore, this multicenter, retrospective, cross-sectional study aimed to determine whether a method using the AcT ratio is useful for estimating stenosis rates.

## Methods

Among the patients who underwent carotid artery ultrasonography at Dokkyo Medical University Hospital, Nara Medical University Hospital, Akiru Municipal Medical Center, and Kita-Harima Medical Center between January 1, 2017 and December 31, 2019, those who underwent either MR angiography, CT angiography, or DSA within 1 month before or after carotid artery ultrasonography for ICA evaluation were included in this study. Patients who underwent ICA stenosis rate evaluations with unknown modalities; patients with insufficient data, such as missing AcT evaluation; patients with a diameter stenosis rate of ≥ 50% in the CCA; patients with multiple stenotic lesions in the ICA that could be observed via carotid artery ultrasonography; and patients with occluded ICA origins were excluded from this study.

The ICA-origin stenosis rate was determined via NASCET using MR angiography, CT angiography, or DSA. All patients underwent carotid artery ultrasonography in the supine position. CCA evaluation was performed using a linear array probe, whereas ICA evaluation was performed using a convex array probe. In accordance with the standard examination method proposed by the Japan Society of Ultrasonics in Medicine and the Japan Academy of Neurosonology [10], the center frequency of the linear array probe was set as ≥ 7 MHz, and the frequency of the convex array probe was set as 1.5–10.0 MHz. PWD of the CCA was performed approximately 2 cm toward the trunk from the carotid bulb, and that of the ICA was performed approximately 1.5–4 cm cranially from the origin. The sample volume was set as half to one-third of the vessel diameter such that the stenosis area was included. In addition, the Doppler incident angle was set within 60 degrees in the blood flow direction, and the sweep speed was evaluated between 6.58 and 60.0 mm/sec.

**Fig. 1:**
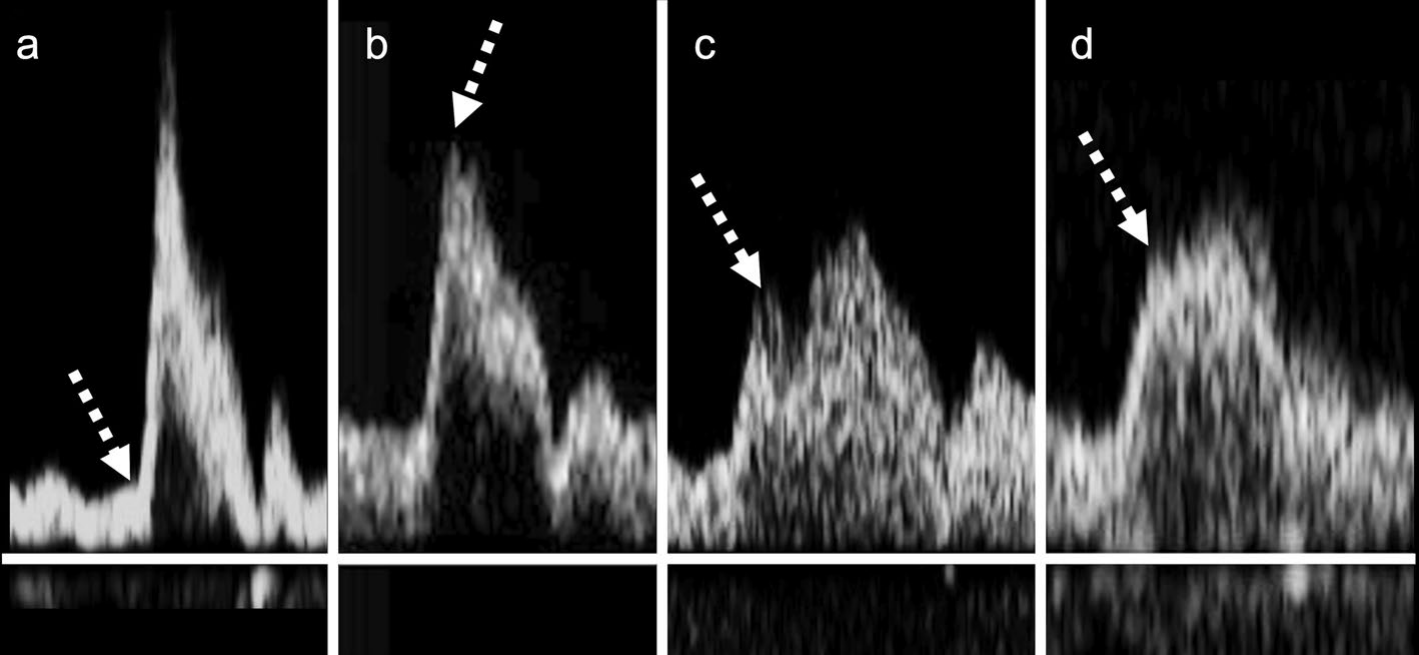
Measurement of AcT. The AcT measurement starting point is the point (**a**) that rises sharply. The time to PSV is measured in cases with no obvious inflection point (**b**). The time to PSV is measured up to the first peak in cases with a bimodal peak pattern (**c**), and up to the inflection point in cases with a clear inflection point (**d**) (dotted arrow). AcT, modified acceleration time; PSV, peak systolic velocity

Figure 1 depicts the evaluation of AcT. The measurement start point was a steeply rising point (Fig. 1a), and the endpoint was a point reported by Takekawa et al. [5, 7–9]. In other words, the time to PSV was taken if the curve was unimodal with no obvious bending point or if the inflection point was unclear (Fig. 1b). In contrast, the time to the first peak was taken if the peak was bimodal (Fig. 1c). The inflection point was defined as the point if there was a clear inflection point before the first peak of a monomodal or bimodal peak pattern (Fig. 1d). The AcT ratio was calculated using the following formula:

$${\text{AcT ratio }} = \, {{\text{AcT of ICA}} \mathord{\left/ {\vphantom {{\text{AcT of ICA}} {\text{AcT of ipsilateral CCA}}}} \right. \kern-0pt} {\text{AcT of ipsilateral CCA}}}$$

AcT is prolonged when there is stenosis [8, 9]. Therefore, if the AcT ratio is less than 1, it is inferred that there is no stenosis in the ICA. Thus, the AcT ratio was set as “AcT ratio = 1” when it was < 1. The presence or absence and severity of aortic stenosis [11] were examined in patients who underwent transthoracic echocardiography (TTE).

### Statistical analysis

The correlation between NASCET stenosis and AcT ratio was evaluated using Pearson's correlation coefficient. Simple regression analysis was used to examine whether NASCET stenosis could be predicted from the AcT ratio. The cut-off value was set using the receiver operating characteristic (ROC) curve, and the sensitivity, specificity, positive predictive value (PPV), negative predictive value (NPV), and accuracy of diagnosis of NASCET stenosis of ≥ 50% and ≥ 70% were calculated using the AcT ratio.

Statistical processing and plotting were performed using IBM SPSS Statics (ver. 28.0, Tokyo, Japan), and p-values of < 0.05 were considered statistically significant.

This study was planned by HT, KS, HH, RO, and DT, and supervised by HT and HH. DT, KS, AT, SK, RO, HI, and HH collected the data, and DT, HT, and KS were in charge of the analysis.

## Results

A total of 520 blood vessels (260 cases) were included in this study. After excluding cases wherein the modality of ICA stenosis evaluation was unknown, vessels had AcT evaluation defects, and the ICA origin was occluded, 461 vessels (mean age of 71.0 ± 12.2 years, 170 males) were analyzed. CCA with a diameter stenosis rate of ≥ 50% or multiple stenotic vessels in the ICA that could be observed via carotid artery ultrasonography were not present in any cases.

MR angiography, CT angiography, and DSA were used to evaluate the presence of stenosis near the ICA origin for 175, seven, and 279 vessels, respectively. NASCET stenosis of ≥ 50% and ≥ 70% near the ICA origin was observed in 84 and 44 vessels, respectively.

### Total vascular results

**Fig. 2:**
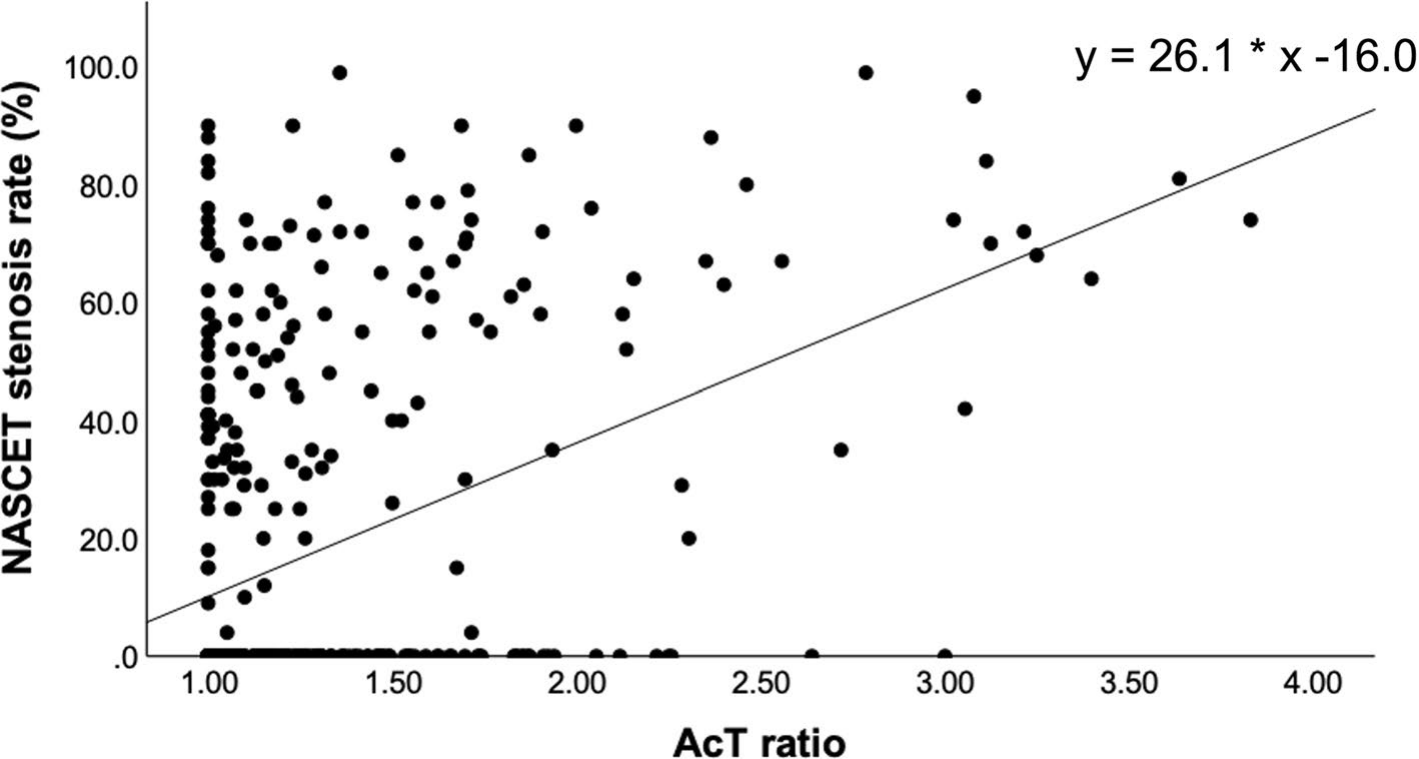
“AcT ratio” and NASCET stenosis. A significant positive correlation is observed between the “AcT ratio” and NASCET stenosis (r = 0.432, p < 0.001, y = 26.1 * × − 16.0) in all 461 vessels. AcT, modified acceleration time; NASCET, North American Symptomatic Carotid Endarterectomy Trial

A significant positive correlation was observed between the AcT ratio and NASCET stenosis (r = 0.432, p < 0.001) (Fig. 2).

**Fig. 3:**
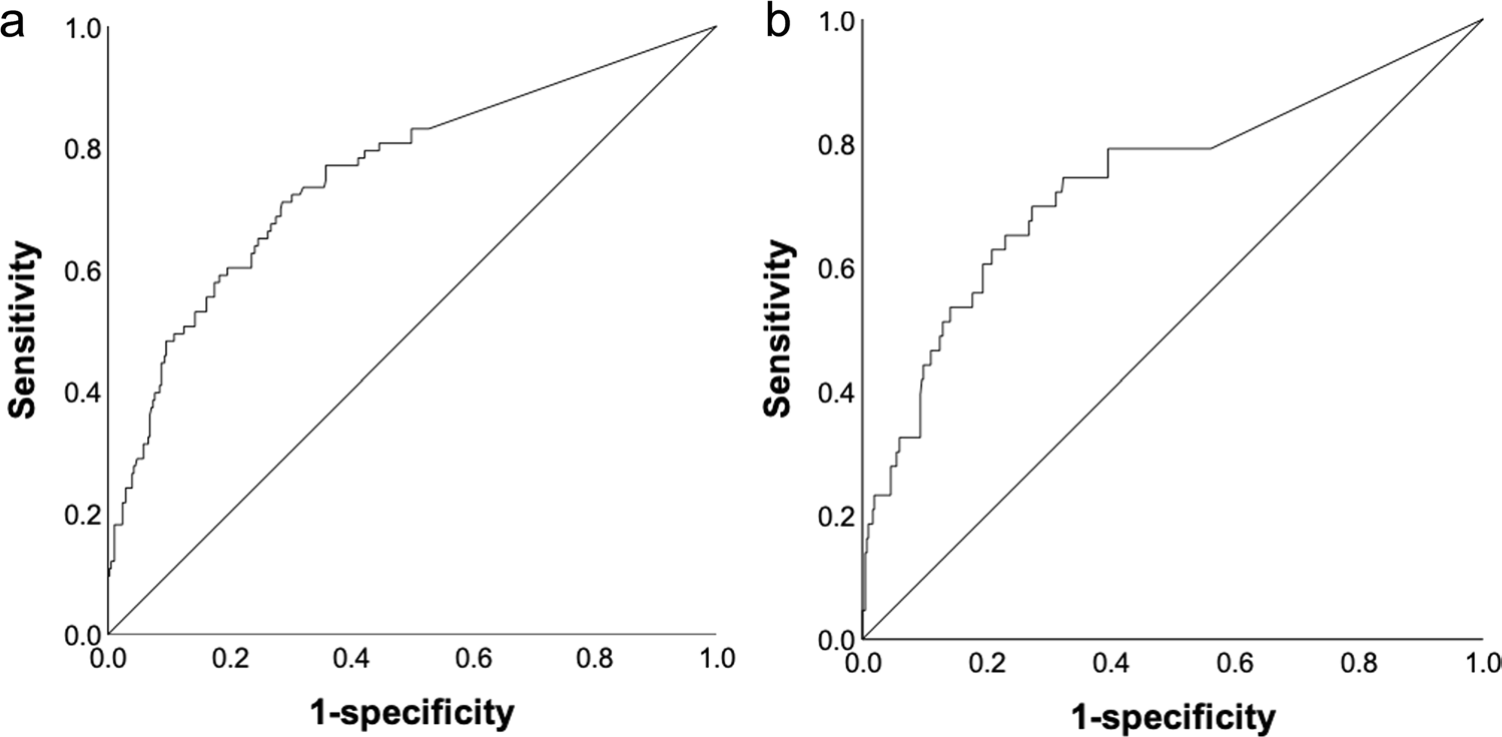
ROC curve for the diagnosis of NASCET stenosis according to the “AcT ratio”. The area under the ROC curve for diagnosing NASCET stenosis of ≥ 50% is 0.757 (**a**), and that for stenosis of ≥ 70% is 0.738 (**b**). ROC, receiver operating characteristic; NASCET, North American Symptomatic Carotid Endarterectomy Trial; AcT, modified acceleration time

Figure 3 presents the results of the ROC curve. The areas under the curve (AUCs) for NASCET stenosis of ≥ 50% and ≥ 70% were 0.757 and 0.738, respectively (Fig. 3a and b). The diagnostic rate of NASCET stenosis of ≥ 50% had a sensitivity, specificity, and accuracy of 71.4%, 70.3%, and 70.5%, respectively, when the cut-off value of the AcT ratio was 1.16. The diagnostic rate had a sensitivity, specificity, and accuracy of 70.2%, 71.6%, and 71.4, respectively, when the cut-off value was 1.17. The NPV was ≥ 91.0% for both cut-off values; however, the PPV was low at around 35% (Table 1).

**Table 1:** Cut-off value of diagnosing NASCET stenosis

	Sensitivity (%)	Specificity (%)	PPV (%)	NPV (%)	Accuracy (%)
Cut-off value for NASCET stenosis ≥ 50%					
< 1.16	71.4	70.3	34.9	91.7	70.5
< 1.17	70.2	71.6	35.5	91.5	71.4
Cut-off value for NASCET stenosis ≥ 70%					
< 1.22	70.5	72.1	21.3	95.9	72.5
< 1.23	65.9	73.1	20.6	95.3	72.5

NASCET, North American Symptomatic Carotid Endarterectomy Trial; PPV, positive predictive value; NPV, negative predictive value

The sensitivity, specificity, and accuracy were 70.5%, 72.1%, and 72.5%, respectively, when the AcT ratio cut-off value for diagnosing a NASCET stenosis of ≥ 70% was 1.22. The sensitivity, specificity, and accuracy were 65.9%, 73.1%, and 72.5%, respectively, when the cut-off value was 1.23. Similar to the cut-off value for diagnosing 50% stenosis, the NPV was high at ≥ 95%; however, the PPV was low at around 21% (Table 1).

### Evaluation results by modality

**Fig. 4:**
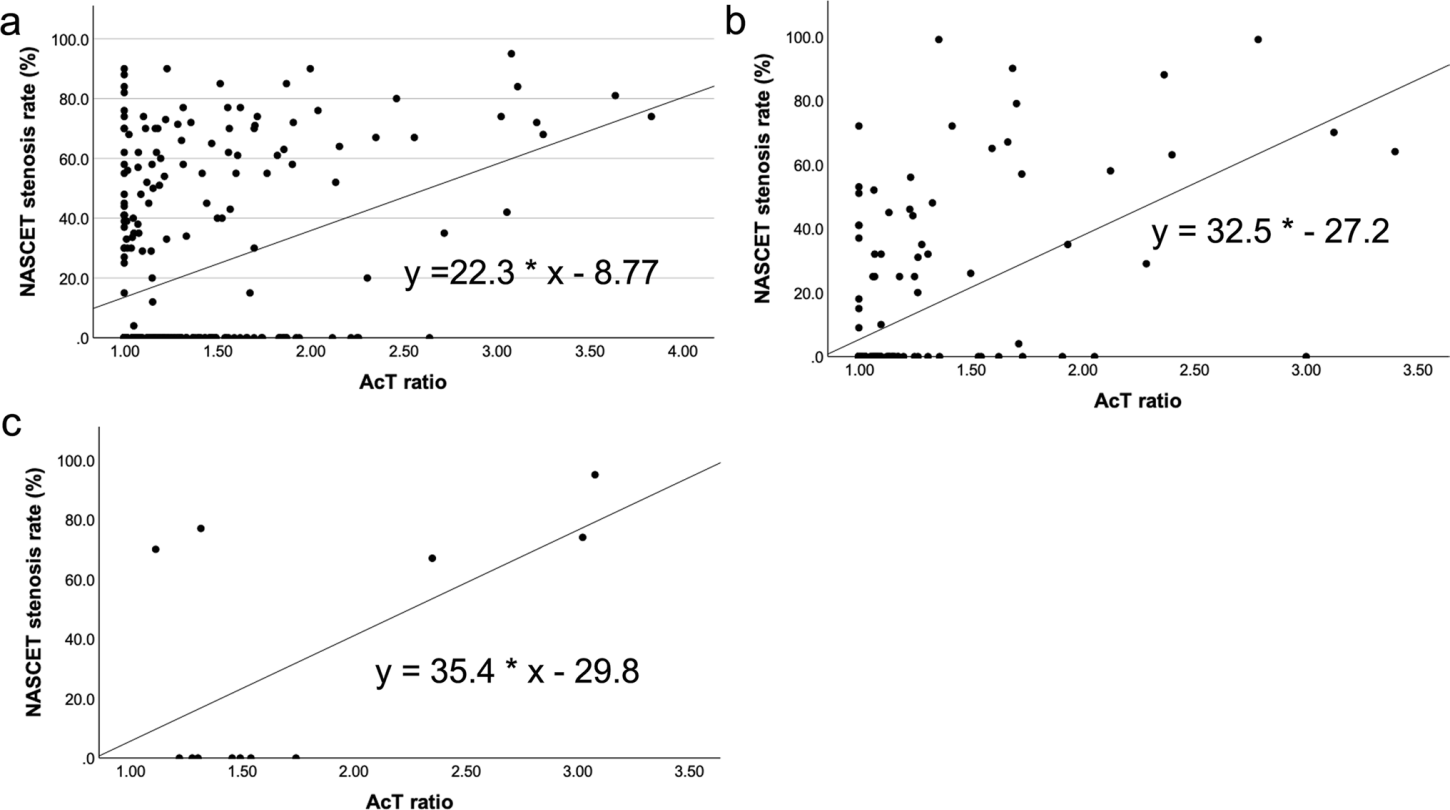
“AcT ratio” and NASCET stenosis based on the stenosis diagnostic methods and aortic stenosis. Both CT angiography and DSA (**a**) (r = 0.364, p < 0.001, y = 22.3 * × − 8.77) and MR angiography (**b**) (r = 0.557, p < 0.001, y = 32.5 * + − 27.2) have a positive correlation with the AcT ratio and NASCET stenosis. A positive correlation is also observed with AS (**c**) (r = 0.611, p = 0.034, y = 35.4 * × + − 29.8). AcT, modified acceleration time; NASCET, North American Symptomatic Carotid Endarterectomy Trial; CT, computed tomography; MR, magnetic resonance

Among the 286 vessels whose NASCET stenosis was determined using CT angiography or DSA, 66 and 36 vessels had stenosis of ≥ 50% and ≥ 70%, respectively. Moreover, a weak but significant positive correlation was observed between CT angiography and AcT ratio (r = 0.364, p < 0.001) (Fig. 4a).

Among the 175 vessels whose NASCET stenosis was determined using MR angiography, 18 and eight vessels had stenosis of ≥ 50% and ≥ 70%, respectively. A significant positive correlation was also observed between MR angiography and AcT ratio (r = 0.557, p < 0.001) (Fig. 4b).

### Aortic stenosis and AcT ratio

Among the 461 vessels analyzed, 332 vessels were analyzed using TTE. Among these 332 vessels, AS was observed in 31 vessels (mild, moderate, and severe AS observed in 19, four, and eight vessels, respectively). All vessels were evaluated with DSA.

Among the 12 vessels with moderate or severe AS, five vessels had NASCET stenosis of ≥ 50%, and four vessels had NASCET stenosis of ≥ 70%. A significant positive correlation was observed between NASCET stenosis and AcT ratio in cases with moderate and severe AS (r = 0.611, p = 0.034) (Fig. 4c).

## Discussion

We conducted a multicenter, retrospective, cross-sectional study to investigate whether AcT ratio, which is the AcT of the ICA divided by the AcT of the ipsilateral CCA, is useful for diagnosing stenosis near the origin of the ICA. The results revealed that the AcT ratio may be useful for estimating NASCET stenosis of ≥ 50%, especially for diagnosis by exclusion, without being influenced by the factors of moderate or severe AS. In other words, an AcT ratio of > 1.17 indicates ≥ 50% stenosis, whereas a cut-off value of > 1.22 indicates ≥ 70% stenosis. Thus, the possibility of the presence of a stenotic lesion is low if the value is smaller than these cut-off values.

The stenosis rate near the ICA origin determined with carotid artery ultrasonography includes the area and diameter stenosis rates determined using B-mode and color or power Doppler imaging. The stenosis rate can be determined using PSV of the stenosis via PWD, and this examination method is widely used [12–14]. A PSV of 125–130 cm/s at the narrowest part of the ICA or a ratio of the narrowest part of the ICA/PSV of the CCA (PSV ratio) ≥ 2 indicates NASCET stenosis of ≥ 50%. A PSV of 200–230 cm/s at the most stenotic part or a PSV ratio of ≥ 4 indicates NASCET stenosis of ≥ 70% [10]. However, since PSV is affected by the Doppler angle of incidence, the stenosis rate may be over- or underestimated depending on the angle of incidence [15].

Moreover it is difficult to measure the PSV at the narrowest part if there is plaque with acoustic shadow, especially circumferential calcified plaque. The presence of turbulence in the blood flow waveform and AT extension evaluated via PWD indicate the presence of a stenotic lesion in such cases [4–6].

Instead of conventional AT that measures from end-diastolic velocity (EDV) to PSV, the measurement start point was set to a point where there is a steep rise in the present study. In addition, the investigation was performed using AcT, a modified AT that measures the time to the inflection point or the first peak. Iizuka et al. [9] compared AT and AcT for 98 blood vessels and reported a significant positive correlation between NASCET stenosis and AcT using DSA; however, no correlation was observed between AT and stenosis rate. The AcT ratio also had a significant positive correlation with the stenosis rate, and the correlation coefficient was superior to AcT. Although AT and AcT are known to be prolonged by AS [16], Okamura et al. reported that the AcT ratio was not affected by the presence of AS after studying 94 blood vessels [17]. Only 12 vessels with moderate or severe AS were included in the present study; however, similar to the study by Okamura et al., the AcT ratio was not affected by AS in the present study, suggesting that it is useful for diagnosing the stenosis rate.

Nishihira et al. [8] investigated the cut-off value of the AcT ratio, which indicates NASCET stenosis of ≥ 50% and ≥ 70% detected using DSA, for 177 vessels in their single-center study. They reported that the AcT ratio was ≥ 1.31 for NASCET stenosis of ≥ 50% and ≥ 1.35 for NASCET stenosis of ≥ 70%. Since the present study evaluated the stenosis rate using MR angiography, CT angiography, and DSA, there may be differences from the report by Nishihira et al. Although the carotid artery stenosis rate differs according to the examination methods and post-processing techniques [18], MR angiography, CT angiography, and DSA are all useful examination methods. In particular, the stenosis rate obtained using contrast-enhanced MR angiography has a high concordance rate with DSA [19]. A significant correlation was observed between AcT ratio and NSACET stenosis detected using MR angiography and CT angiography/DSA in the present study. However, only cases with the stenosis rate evaluated within 1 month before and after carotid artery ultrasonography were included in the present study. Carotid artery plaque may change within a month, depending on its nature and the drugs prescribed [20]. Data regarding the period between carotid artery ultrasonography and stenosis rate was not collected in the present study. The study by Nishihira et al. focused on patients with a history of stroke, and the period between carotid ultrasonography and DSA may be shorter than that in the present study. Furthermore, it was not possible to collect data on therapeutic drugs. Thus, it is possible that the cut-off value of the AcT ratio for suspecting stenosis was different than that in the study by Nishihira et al.

This study had some limitations. Since this was a retrospective study, the Doppler incident angle during PWD measurement and the AcT evaluation site for CCA and ICA could not be standardized. Similarly, whether AcT was measured using one heartbeat or the average of multiple heartbeats could not be standardized. Furthermore, the curvature of the ICA and the length of the stenotic lesion could not be evaluated, which may have affected AcT. Although the extent to which these factors affect AcT is unclear as no similar studies have been conducted, this is a factor that should be investigated in the future. Furthermore, in addition to the lack of uniformity in image evaluation for diagnosing stenosis rate, other limitations, such as the inability to investigate the effects of valvular heart disease and heart disease other than AS (as TTE was not performed in all patients), were also present. In addition, we regarded the AcT ratio as 1 when it was less than 1, which was different from the actual value. A prospective study that considers these factors and sets a shorter period for carotid artery ultrasonography and stenosis rate evaluation is necessary.

However, no multicenter study has reported on the usefulness of the AcT ratio. The present study demonstrates the usefulness of this examination method in estimating the presence or absence of stenotic lesions in cases where PSV at the stenotic site cannot be measured directly.

## Conclusion

AcT ratio, which is defined as the AcT of the ICA divided by the AcT of the ipsilateral CCA, was shown to be useful for diagnosing stenosis near the origin of the ICA. NASCET stenosis of ≥ 50% was considered unlikely if the AcT ratio was ≤ 1.17, whereas NASCET stenosis of ≥ 70% was considered unlikely if it was ≤ 1.22.An AcT ratio cut-off value of 1.17 indicates NASCET stenosis of ≥ 50%, whereas a cut-off value of 1.22 indicates NASCET stenosis of ≥ 70%.

## References

[1] Rothwell PM, Gibson RJ, Slattery J (1994). Prognostic value and reproducibility of measurements of carotid stenosis. A comparison of three methods on 1001 angiograms. European Carotid Surgery Trialists' Collaborative Group. Stroke..

[2] Cui L, Han Y, Zhang S (2018). Safety of stenting and endarterectomy for asymptomatic carotid artery stenosis: a meta-analysis of randomised controlled trials. Eur J Vasc Endovasc Surg.

[3] Barnett HJM, Taylor DW, Haynes RB, North American Symptomatic Carotid Endarterectomy Trial Collaborators (1991). Beneficial effect of carotid endarterectomy in symptomatic patients with high-grade carotid stenosis. N Engl J Med..

[4] Takekawa H, Tsukui D, Kobayasi S (2022). Ultrasound diagnosis of carotid artery stenosis and occlusion. J Med Ultrason.

[5] Takekawa H, Asakawa Y, Lee T (2009). Usefulness of acceleration time for assessment of stenosis in the extracranial internal carotid artery. Neurosonology.

[6] Tamura H, Akaiwa Y, Onda K (2013). Usefulness of acceleration time for internal carotid artery origin stenosis. Ann Vasc Dis.

[7] Takekawa H, Suzuki K, Takada E (2014). Acceleration time ratio for the assessment of extracranial internal carotid artery stenosis. J Med Ultrason.

[8] Nishihira T, Takekawa H, Suzuki K (2018). Usefulness of accel- eration time ratio in diagnosis of internal carotid artery origin stenosis. J Med Ultrason.

[9] Iizuka K, Takekawa H, Iwasaki A (2020). Suitable methods of measuring acceleration time in the diagnosis of internal carotid artery stenosis. J Med Ultrason.

[10] The Japan society of ultrasonics in medicine. Standard methods for the evaluation of carotid artery lesions by ultrasound. 2017. https://www.jsum.or.jp/committee/diagnostic/pdf/jsum0515_guideline.pdf. Accessed 14 Sep 2023.

[11] Baumgartner H, Hung J, Bermejo J (2017). Recommendations on the echocardiographic assessment of aortic valve stenosis: a focused update from the european association of cardiovascular imaging and the American Society of Echocardiography. J Am Soc Echocardiogr.

[12] Koga M, Kimura K, Minematsu K (2001). Diagnosis of internal carotid artery stenosis greater than 70% with power Doppler duplex sonography. AJNR Am J Neuroradiol.

[13] AbuRahma AF, Srivastava M, Stone PA (2011). Critical appraisal of the Carotid Duplex Consensus criteria in the diagnosis of carotid artery stenosis. J Vasc Surg..

[14] Tokunaga K, Koga M, Yoshimura S (2016). Optimal peak systolic velocity thresholds for predicting internal carotid artery stenosis greater than or equal to 50%, 60%, 70%, and 80%. J Stroke Cerebrovasc Dis.

[15] Tola M, Yurdakul M (2006). Effect of Doppler angle in diagnosis of internal carotid artery stenosis. J Ultrasound Med.

[16] O’Boyle MK, Vibhakar NI, Chung J (1996). Duplex sonography of the carotid arteries in patients with isolated aortic stenosis: imaging findings and relation to severity of stenosis. AJR Am J Roentgenol.

[17] Okamura M, Takekawa H, Suzuki K (2013). Evaluation of factors that prolong acceleration time of the common and internal carotid arteries. Neurosonology.

[18] Lell M, Fellner C, Baum U (2007). Evaluation of carotid artery stenosis with multisection CT and MR imaging: influence of imaging modality and postprocessing. AJNR Am J Neuroradiol.

[19] Alvarez-Linera J, Benito-León J, Escribano J (2003). Prospective evaluation of carotid artery stenosis: elliptic centric contrast-enhanced MR angiography and spiral CT angiography compared with digital subtraction angiography. AJNR Am J Neuroradiol.

[20] Hirano M, Nakamura T, Kitta Y (2009). Rapid improvement of carotid plaque echogenicity within 1 month of pioglitazone treatment in patients with acute coronary syndrome. Atherosclerosis.

